# Calcineurin-Inhibitor Minimization in Liver Transplant Patients with Calcineurin-Inhibitor-Related Renal Dysfunction: A Meta-Analysis

**DOI:** 10.1371/journal.pone.0024387

**Published:** 2011-09-09

**Authors:** Yuan Kong, Dongping Wang, Yushu Shang, Wenhua Liang, Xiaoting Ling, Zhiyong Guo, Xiaoshun He

**Affiliations:** Organ Transplant Center, First Affiliated Hospital, Sun Yat-sen University, Guangzhou, China; University of Southern California, United States of America

## Abstract

**Background:**

Introduction of calcineurin-inhibitor (CNI) has made transplantation a miracle in the past century. However, the side effects of long-term use of CNI turn out to be one of the major challenges in the current century. Among these, renal dysfunction attracts more and more attention. Herein, we undertook a meta-analysis to evaluate the efficacy and safety of calcineurin-inhibitor (CNI) minimization protocols in liver transplant recipients with CNI-related renal dysfunction.

**Methods:**

We included randomized trials with no year and language restriction. All data were analyzed using random effect model by Review Manager 5.0. The primary endpoints were glomerular filtration rate (GFR), serum creatinine level (sCr) and creatinine clearance rate (CrCl), and the secondary endpoints were acute rejection episodes, incidence of infection and patient survival at the end of follow-up.

**Results:**

GFR was significantly improved in CNI minimization group than in routine CNI regimen group (Z = 5.45, *P*<0.00001; I^2^ = 0%). Likely, sCr level was significantly lower in the CNI minimization group (Z = 2.84, *P* = 0.005; I^2^ = 39%). However, CrCl was not significantly higher in the CNI minimization group (Z = 1.59, *P* = 0.11; I^2^ = 0%). Both acute rejection episodes and patient survival were comparable between two groups (rejection: Z = 0.01, *P* = 0.99; I^2^ = 0%; survival: Z = 0.28, *P* = 0.78; I^2^ = 0%, respectively). However, current CNI minimization protocols may be related to a higher incidence of infections (Z = 3.06, *P* = 0.002; I^2^ = 0%).

**Conclusion:**

CNI minimization can preserve or even improve renal function in liver transplant patients with renal impairment, while sharing similar short term acute rejection rate and patient survival with routine CNI regimen.

## Introduction

With 1-year liver allograft survival rates now exceeding 80% [1], attention is increasingly being paid on improving long-term morbidity and mortality in liver transplant recipients. Renal dysfunction is the most concerned long-term complication post-liver transplantation, because it was estimated that 18% of recipients would develop chronic renal failure or end-stage renal disease by 5 years post-transplant and renal dysfunction would significantly increase mortality risk [2]–[4].

Multiple factors have been reported to be involved in chronic renal impairment in liver transplant recipients [2], [4], [5]. Among these, high level exposure of calcineurin-inhibitors (CNI), namely cyclosporin A (CsA) and tacrolimus (Tac), is a well recognized risk factor [2], [6], [7]. Importantly, although chronic CNI-induced renal insufficiency is associated with structural changes in the kidney [8]–[10], an improvement in renal function can be observed in patients after CNI reduction [11]–[13]. Nonetheless, the initial attempts to withdraw CNI leaded to increased acute rejection risk [14]. To tip the balance between potent immunosuppression and less CNI exposure, several prospective, randomized, and controlled trials (RCTs) of novel CNI minimization protocols were conducted recently [15]–[23]. However, current knowledge about these protocols is dependent on single institution studies, which was often limited by small sample sizes and individual practice patterns.

Herein, we performed a meta-analysis of the available literature to better understand the efficacy and safety of CNI minimization protocols in liver transplant patients with CNI-related renal dysfunction. This data provide important insight capable of better informing clinical physicians regarding the treatment of CNI-related renal dysfunction.

## Methods

### Study design, search strategy, and study selection

Before data collection, two general protocols were designed to be compared: CNI minimization regimen and routine CNI regimen. To limit publication bias, we included published trials with no language or year restrictions. Initial searches of MEDLINE, EMBASE databases and the Cochrane Database of Systematic Reviews included terms: calcineurin-inhibitor, cyclosporin A, tacrolimus or FK506, minimization, withdrawal, reduction, elimination and liver transplantation.

To be included, trials had to be randomized, not confounded by additional therapeutic differences between the two protocols. Trials should compare renal function of liver transplant recipients receiving routine CNI regimen versus CNI minimization regimen for CNI-related renal impairment. To limit the renal function in a comparable range, we only included patients with glomerular filtration rate (GFR) under 60 ml/min, serum creatinine level (sCr) more than 1.5 mg/dl or creatinine clearance rate (CrCl) under 70 ml/min before enrollment according to the National Kidney Foundation (NKF) recommendation for chronic kidney disease (CKD) and the staging index used in clinical practice. To make sure that routine CNI regimen and CNI minimization protocols are practiced as their names, in each trial the CNI dose in the minimization group should be initially reduced by at least 25% of the dose as is used in the routine regimen group to achieve a lower target trough levels, or CNI were completely withdrawn and converted to non-CNI based protocols.

### Quality assessment of trials included

A quality assessment was carried out for all the retrieved RCTs. Quality in a systematic review essentially refers to the absence of biases. To assess the methodological validity of the studies included in this review the following aspects were evaluated: allocation concealment, intention to treat analysis or not, blinding and description of handling of missing data. Articles were assessed by two reviewers (DW and YS) independently. Disagreements were resolved by consultation with a third reviewer (ZG).

### Data extraction and outcome measures

For the trials included in our meta-analysis, we sought data for demography information, renal function (GFR, sCr and CrCl), acute rejection (AR), incidence of infections (including cytomegalovirus (CMV), varicella zoster virus (VZV), herpes simplex infection and nasopharyngitis, bronchitis, pneumonia, stomatitis events and urinary tract infection (UTI)), and patient survival for all patients. The primary outcomes of our meta-analysis were renal function, and the secondary outcomes were AR, incidence of various infections and patient survival. The data were extracted by two investigators (YK and YS) independently. The conduct and reporting were in accordance with the Quality of Reporting of Meta-Analyses statement.

### Statistical analysis

For every outcome, we used the statistical software Review Manager 5.0 (The Cochrane Collaboration, Oxford, United Kingdom) to analyze the collected data and to compare each treatment group with the routine CNI regimen group. The primary outcomes (GFR, sCr and CrCl) and the secondary outcomes (AR, incidence of infections, patient survival) were analyzed as continuous and dichotomized variables using random effect model, and their results were reported as mean difference (95% confidence interval) and odds ratio (95% confidence interval), respectively. The statistic strength was measured by overall effect size Z and heterogeneity index I^2^.

## Results

### Characteristics of included studies

**Figure 1:**
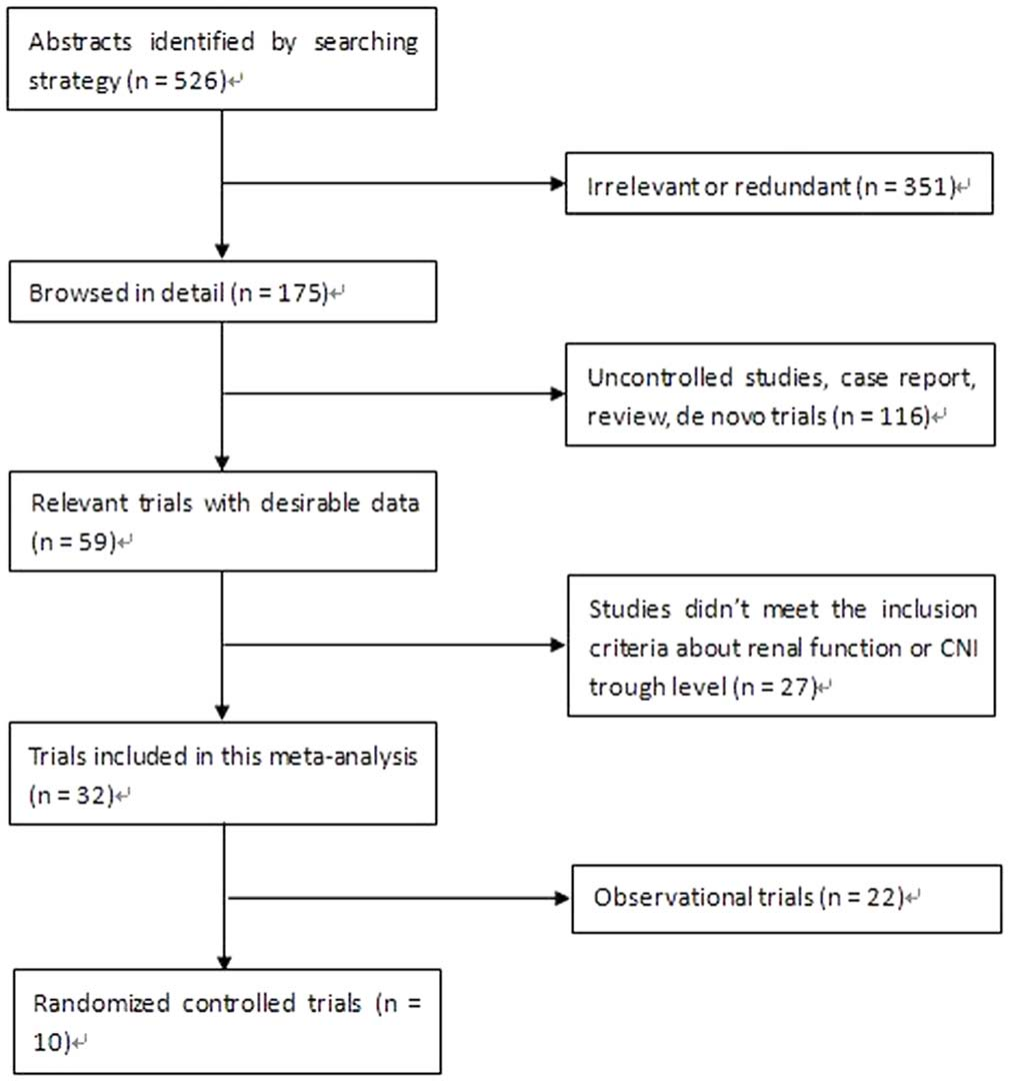
Flow diagram of study identification.

We included 32 trials with 1383 patients in the current meta-analysis, including 10 RCTs [14]–[23] with 625 patients and 22 observational trials [12], [13], [24]–[43] with 758 patients. Figure 1 shows the flow diagram of study identification. Half of the 10 RCTs achieved CNI withdrawal or completely conversion in the end of study [14], [15], [19], [20], [22]. For those CNI was not completely withdrawn, MMF was started mostly at 500 mg twice a day [16]–[18], [23] and in one study at 1000 mg twice a day [21] and eventually achieved 1000–2000 mg twice a day. Accordingly, CNI dose was gradually reduced by at least 25% to reach CsA trough level of 25–50 ng/mL or Tac trough level of 2–4 ng/mL [16]–[18], [23]. Since the included 32 trials used different measures of primary outcome, we analyzed the data according to 3 different outcome measures: GFR, sCr and CrCl. Additionally, 22 observational trials compared renal function collected at baseline pre- and post-conversion. Although it was inappropriate to combine these data in a meta-analysis with that of 10 RCTs, considering these trials did follow our principle of this analysis, we included them anyway and did a separate meta-analysis for reference and supplement. Table 1 shows the basic characteristics of the included studies (the first 10 were RCTs and the remaining 22 were observational studies). In general, the CNI minimization protocols used in these 32 trials were divided into 3 categories: mycophenolate mofetil (MMF) -based, sirolimus (SRL) -based and everolimus (ERL) -based regimens. We compared them both individually and collectively with the routine CNI regimen in this meta-analysis to limit heterogeneity and gain a better understanding of the efficacy and safety profiles of different protocols.

**Table 1:** Basic characteristics of the studies included in this meta-analysis.

Author	Year	Country	Number of Patient	Gender (Male/Female)	Drug Used (CNI minimization/Routine CNI regimen)	Initial CNI trough levels (ng/mL)	Duration of Follow-up
H.J.Schlitt et al^14^	2001	Germany	28	19/9	MMF/CNI+AZA+steroids	CsA 100–120/Tac 8–10	6 m
P.De Simone et al^15^	2009	Italy	145	85/60	Everolimus+CNI↓+steroids/CNI*	CsA 105.5/Tac 5.65	12 m
V.R.Cicinnati et al^16^	2007	Germany	75	51/24	CNI↓+MMF+steroids/CNI*	CsA 110.6/Tac 6.59	12 m
S.Beckebaum et al^17^	2004	Germany	32	22/10	CNI↓+MMF/CNI*	CsA 116/Tac 5.6	12 m
G.P.Pageaux et al^18^	2006	France	56	45/11	CNI↓+MMF/CNI*	CsA 162/Tac 4.4	12 m
U.Eisenberger et al^19^	2009	Switzerland	16	12/4	Sirolimus*/CNI*	CsA 160/Tac 10/8	12 m
C.C.Rogers et al^20^	2009	U.S.A.	82	46/36	Sirolimus*/CNI*	CsA 100–150/Tac 6–8	12 m
M.Biselli et al^21^	2009	Italy	60	50/10	CNI↓+MMF/CNI*	CsA 120/Tac 7	12 m
S.Shenoy et al^22^	2007	U.S.A.	40	29/11	Sirolimus*/CNI*	CsA 150/Tac 6.3	12 m
S.Beckbaum et al^23^	2009	Germany	90	63/27	CNI↓+MMF/CNI*	N/A	12 m
A.Kornberg et al^12^	2005	Germany	43	29/14	CNI↓+MMF/CNI*	CsA 143.6/Tac 9.9	6 m
M.Cantarovich et al^13^	2003	Canada	19	N/A	CsA↓+MMF/CsA*	CsA 132	12 m
L.B.Pulido et al^24^	2008	Spain	31	N/A	MMF*/CNI*	CsA 65.63/Tac 3.72	12 m
D.Reich et al^25^	2005	U.S.A.	15	10/5	MMF+steroids/CNI*	CsA 188.8/Tac 10/8	13 m
F.Di Benedetto et al^26^	2009	Italy	31	N/A	Sirolimus+steroids/CNI+steroids	N/A	36 m
C.Ponton et al^27^	2010	Spain	88	74/14	CNI↓+MMF/CNI*	N/A	6 m
C.Creput et al^28^	2007	France	49	37/12	CNI↓+MMF/CNI+AZA+steroids	CsA 100–250/Tac 5–10	36 m
R.O.Koch et al^29^	2004	Austria	32	22/10	CNI↓+MMF+steroids/CNI+AZA+steroids	CsA 32/Tac 2.7	6 m
M.L.Raimondo et al^30^	2003	U.K.	16	10/6	MMF*/CNI+AZA+steroids	N/A	12 m
J.M.M.Planas et al^31^	2004	Spain	50	32/18	MMF/CNI*	CsA 93/Tac 6.5	18 m
U.Tannuri et al^32^	2007	Brazil	11	5/6	CNI↓+MMF/CNI+ steroids	Tac 6–8	24 m
R.Pfitzmann et al^33^	2002	Germany	47	N/A	CNI↓+MMF/CNI+steroids	CsA 100–180/Tac 5–10	6 m
K.D.Fairbanks et al^34^	2003	U.S.A.	21	10/11	Sirolimus*/CNI*	N/A	16 m
E.Q.Sanchez et al^35^	2005	U.S.A.	35	N/A	Sirolimus+CNI↓+MMF/CNI+MMF+steroids	N/A	24 m
Y.J.Yang et al^36^	2008	China	16	16/0	Sirolimus*/CNI*	N/A	6 m
G.Orlando et al^37^	2007	Italy	42	34/8	MMF/CNI*	CsA 129/Tac 2.3	12 m
P.De Simone et al^38^	2009	Italy	70	51/19	Everolimus*/CNI*	CsA 100–150/Tac 3–8	12 m
S.Dharancy et al^39^	2009	France	52	43/9	MMF*/CNI+AZA+steroids	CsA 150–250/Tac 6–12	12 m
I.Morard et al^40^	2007	Switzerland	9	N/A	Sirolimus*/CNI*	CsA 96/Tac 7	23 m
M.Vivarelli et al^41^	2010	Italy	28	N/A	Sirolimus+steroids/CNI*	N/A	12 m
J.Castroagudin et al^42^	2011	Spain	30	26/4	Everolimus*/CNI*	N/A	12 m
A.H.Cotterell et al^43^	2002	U.S.A.	8	5/3	Sirolimus+CNI↓/CNI*	N/A	12 m

*Regimen based on one drug with concomitant drug(s) that was either prednisone, or AZA, or MMF. Abbreviations: CNI, calcineurin inhibitor; MMF, mycophenolate mofetil; AZA, azathioprine; N/A, not available.

### Methodological quality

In general, the methodological quality of the included 10 RCTs was not bad. However, only 3 studies described the allocation sequences. And 3 studies used the intention-to-treat analysis to avoid detection and attrition bias. All RCTs except one were open-labeled study. Table 2 summarizes the risk evaluation of bias.

**Table 2:** Risk of bias in the randomized controlled trials.

Authors	Allocation Sequence Described	Intention To Treat Analysis	Blinding			Handling Of Missing Data
			Patient	Personnel	Assessor	
Schlitt et al (2001)^14^	No	Yes	Yes	No	No	Unclear
Simone et al (2009)^15^	Yes	Yes	No	No	No	Unclear
Cicinnati et al (2007)^16^	No	Yes	No	No	No	Unclear
Beckebaum et al (2004)^17^	No	No	No	No	No	Unclear
Pageaux et al (2006)^18^	No	No	No	No	No	Unclear
Eisenberger et al (2009)^19^	Yes	No	No	No	No	Unclear
Rogers et al (2009)^20^	No	No	No	No	No	Unclear
Biselli et al (2009)^21^	No	No	No	No	No	Unclear
Shenoy et al (2007)^22^	Yes	No	No	No	No	Last Value Forward
Beckbaum et al (2009)^23^	No	No	No	No	No	Unclear

### CNI minimization improves renal function

**Figure 2:**
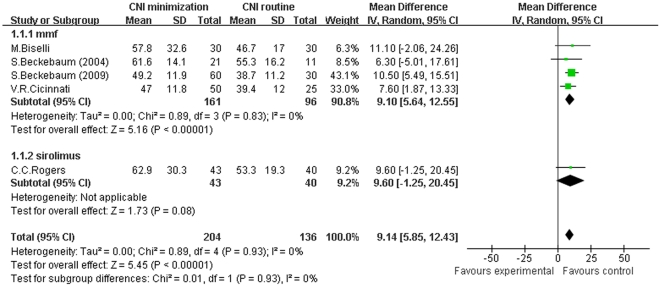
Meta-analysis of CNI minimization versus routine CNI regimen in GFR (RCTs). In MMF subgroup, the GFR of recipients was significantly higher than in routine CNI regimen group. In sirolimus subgroup, the improvement of GFR over routine CNI regimen group was not statistically significant. In the total 10 RCTs, the GFR was significantly improved. Z = total effect size, I^2^ = heterogeneity index. Columns in green represent the mean difference of each study and column size represents the study weight in the analysis. Lanes represent the 95% CI of each study. Diamonds in black represent the overall effect size and diamond width represents the overall 95% CI.

Firstly, we conducted a meta-analysis of 10 RCTs regarding GFR in patients receiving CNI minimization (including MMF and sirolimus subgroups) versus routine CNI regimen, which was shown in Figure 2. The forest plot graph of the comparison showed that in MMF subgroup, GFR of recipients was significantly higher than in routine CNI regimen group (Z = 5.16, *P*<0.00001; I^2^ = 0%). While in sirolimus subgroup, the improvement of GFR over routine CNI regimen group was not statistically significant (Z = 1.73, *P* = 0.08; I^2^ not applicable). In total, in the included RCTs, GFR was significantly improved in CNI minimization group as compared to routine CNI regimen group (Z = 5.45, *P*<0.00001; I^2^ = 0%). Similarly, a meta-analysis of the included observational trials regarding GFR (shown in Figure S1) demonstrated that in MMF subgroup, GFR was significantly higher than in the routine CNI regimen group (Z = 3.95, *P*<0.0001; I^2^ = 71%). And in sirolimus subgroup, the improvement of GFR over the routine CNI regimen group was also significant (Z = 3.17, *P* = 0.002; I^2^ = 90%). Collectively, in the included observational studies, GFR was significantly improved in the CNI minimization group (Z = 3.59, *P* = 0.0003; I^2^ = 94%).

**Figure 3:**
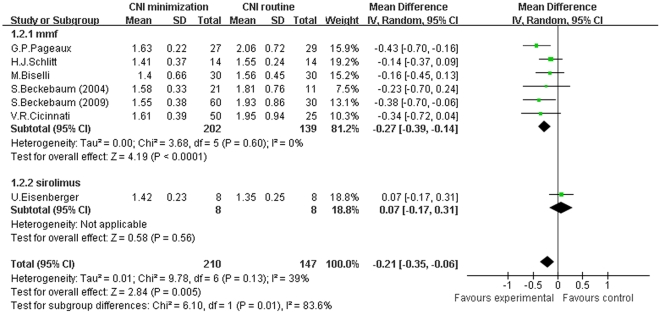
Meta-analysis of CNI minimization versus routine CNI regimen in sCr (RCTs). In MMF subgroup, the sCr level was significantly lower than in routine CNI regimen group. In sirolimus subgroup, the decrease of sCr level was not statistically significant. In the total 10 RCTs, the sCr level was significantly decreased in CNI minimization group. Z = total effect size, I^2^ = heterogeneity index. Columns in green represent the mean difference of each study and column size represents the study weight in the analysis. Lanes represent the 95% CI of each study. Diamonds in black represent the overall effect size and diamond width represents the overall 95% CI.

Then, we conducted a meta-analysis of sCr of patients receiving CNI minimization (including MMF and sirolimus subgroups) versus routine CNI regimen, which was shown in Figure 3. The forest plot graph of the comparison showed that in MMF subgroup, the sCr level of patients was significantly lower than in routine CNI regimen group (Z = 4.19, *P*<0.0001; I^2^ = 0%). While in sirolimus subgroup, the decrease of sCr level was not statistically significant in comparison with routine CNI regimen group (Z = 0.58, *P* = 0.56; I^2^ not applicable). Collectively, in the included RCTs, the sCr level was significantly decreased in CNI minimization group (Z = 2.84, *P* = 0.005; I^2^ = 39%). In addition, a meta-analysis of the included observational trials regarding sCr level (Figure S2) showed that in MMF subgroup, the sCr level was significantly lower than in routine CNI regimen group (Z = 6.76, *P*<0.00001; I^2^ = 82%). And in sirolimus subgroup, there was a significant decrease of sCr level compared to routine CNI regimen group (Z = 7.91, *P*<0.00001; I^2^ = 56%). And in everolimus subgroup, the sCr level was also significantly decreased (Z = 2.68, *P* = 0.007; I^2^ not applicable). Totally, in the included observational studies, the sCr level was significantly decreased in CNI minimization group (Z = 6.63, *P*<0.00001; I^2^ = 91%).

**Figure 4:**
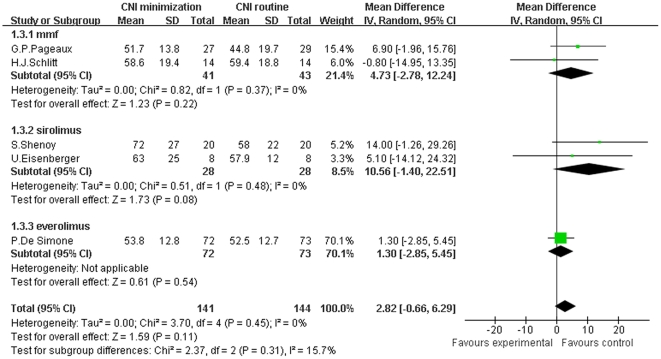
Meta-analysis of CNI minimization versus routine CNI regimen in CrCl (RCTs). In MMF, sirolimus and everolimus subgroup, the improvement of CrCl over routine CNI regimen group was not statistically significant, as well as in the total 10 RCTs. Z = total effect size, I^2^ = heterogeneity index. Columns in green represent the mean difference of each study and column size represents the study weight in the analysis. Lanes represent the 95% CI of each study. Diamonds in black represent the overall effect size and diamond width represents the overall 95% CI.

Finally, we conducted a meta-analysis of CrCl of patients receiving CNI minimization (including MMF, sirolimus and everolimus subgroups) versus routine CNI regimen, which was shown in Figure 4. The forest plot graph of the comparison showed that in MMF subgroup, the improvement of CrCl over routine CNI regimen group was not statistically significant (Z = 1.23, *P* = 0.22; I^2^ = 0%). And in sirolimus subgroup, once again, we could not document a significant improvement of CrCl (Z = 1.73, *P* = 0.08; I^2^ = 0%), neither did we in everolimus subgroup (Z = 0.61, *P* = 0.54; I^2^ not applicable). In total, in the included RCTs, CrCl was not significantly improved in CNI minimization group over routine CNI regimen group (Z = 1.59, *P* = 0.11; I^2^ = 0%). In contrast, a meta-analysis of the included observational trials regarding CrCl (Figure S3) showed that in MMF subgroup, CrCl was significantly higher than in routine CNI regimen group (Z = 3.69, *P* = 0.0002; I^2^ = 80%). While in everolimus subgroup, improvement of CrCl over routine CNI regimen group was not significant (Z = 1.88, *P* = 0.06; I^2^ not applicable). Totally, in the included observational studies, CrCl was significantly improved in CNI minimization group (Z = 4.02, *P*<0.0001; I^2^ = 75%).

### CNI minimization does not compromise short term acute rejection and patient survival, but increases infection rates

To evaluate the safety of CNI minimization protocols, we then compared acute rejection episodes, incidence of infections and patient survival between CNI minimization and routine CNI regimen group.

**Figure 5:**
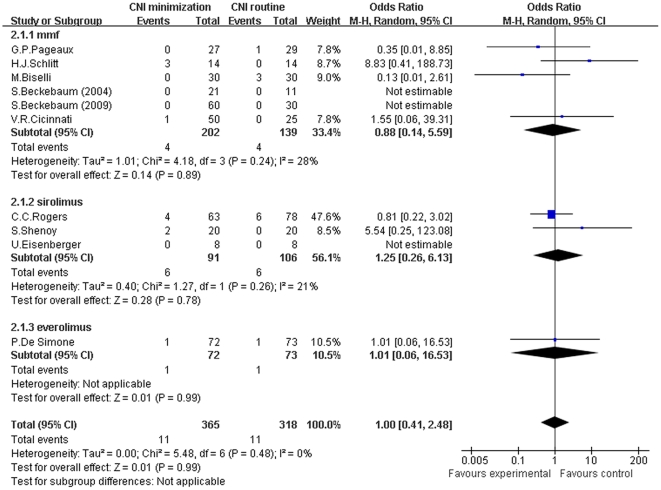
Meta-analysis of CNI minimization versus routine CNI regimen in acute rejection (RCTs). There was no significant difference between CNI minimization and routine CNI regimen group in all subgroup and total analysis. Z = total effect size, I^2^ = heterogeneity index. Columns in blue represent the odds ratio of each study and column size represents the study weight in the analysis. Lanes represent the 95% CI of each study. Diamonds in black represent the overall effect size and diamond width represents the overall 95% CI.

For the meta-analysis of CNI minimization versus routine CNI regimen in acute rejection, there was no significant difference between two groups in all subgroups and total analysis (MMF subgroup: Z = 0.14, *P* = 0.89; I^2^ = 28%; sirolimus subgroup: Z = 0.28, *P* = 0.78; I^2^ = 21%; everolimus subgroup: Z = 0.01, *P* = 0.99; I^2^ not applicable; and total: Z = 0.01, *P* = 0.99; I^2^ = 0%) (shown in Figure 5).

**Figure 6:**
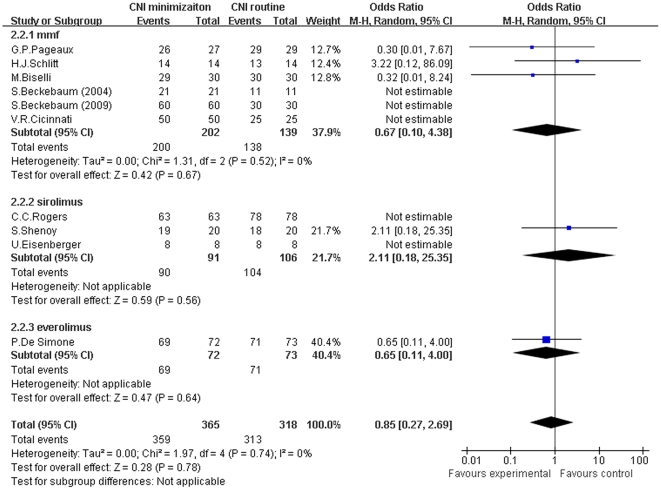
Meta-analysis of CNI minimization versus routine CNI regimen in infection incidence (RCTs). In MMF and everolimus subgroup, the difference of infection incidence was not statistically significant. In sirolimus subgroup, the infection incidence was significantly higher than in routine CNI regimen group. In total, the infection incidence was significantly higher in CNI minimization group. Z = total effect size, I^2^ = heterogeneity index. Columns in blue represent the odds ratio of each study and column size represents the study weight in the analysis. Lanes represent the 95% CI of each study. Diamonds in black represent the overall effect size and diamond width represents the overall 95% CI.

We also conducted a meta-analysis of the incidence of various infections between two groups (shown in Figure 6). The difference of incidence of infections between MMF/everolimus subgroup and routine CNI regimen group was comparable (Z = 1.96, *P* = 0.05; I^2^ = 0%; Z = 1.36, *P* = 0.18; I^2^ not applicable). While in sirolimus subgroup, the infection incidence was significantly higher than in routine CNI regimen group (Z = 2.02, *P* = 0.04; I^2^ = 0%). In total, the infection incidence was significantly higher in CNI minimization group (Z = 3.06, *P* = 0.002; I^2^ = 0%).

**Figure 7:**
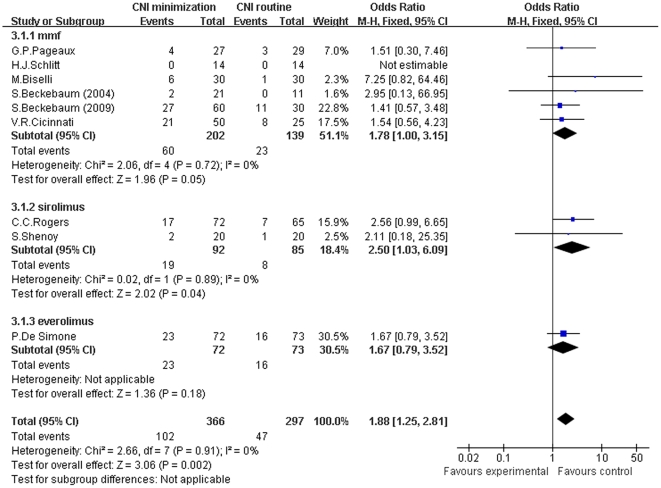
Meta-analysis of CNI minimization versus routine CNI regimen in patient survival (RCTs). There was no significant difference between CNI minimization and routine CNI regimen group in all subgroup and total analysis. Z = total effect size, I^2^ = heterogeneity index. Columns in blue represent the odds ratio of each study and column size represents the study weight in the analysis. Lanes represent the 95% CI of each study. Diamonds in black represent the overall effect size and diamond width represents the overall 95% CI.

For the meta-analysis of patient survival at the end of the follow-up, there was no significant difference between CNI minimization and routine CNI regimen group in all subgroups and in total (MMF subgroup: Z = 0.42, *P* = 0.67; I^2^ = 0%; sirolimus subgroup: Z = 0.59, *P* = 0.56; I^2^ not applicable; everolimus subgroup: Z = 0.47, *P* = 0.64; I^2^ not applicable; and total: Z = 0.28, *P* = 0.78; I^2^ = 0%) (shown in Figure 7).

## Discussion

CNI provide potent immunosuppression for solid organ transplant patients, however, simultaneously exhibit nephrotoxicity as a major side effect. CNI cause both acute (functional) nephrotoxicity and chronic (structural) nephrotoxicity. Whereas acute nephrotoxicity is reversible by withdrawal of the CNI, chronic nephrotoxicity due to CNIs is thought to be irreversible and even progressive [44]. Withdrawal of CNI during early stages of renal dysfunction results in improvement of renal function when pathologic changes are still reversible [11]–[13]. The principle of CNI minimization protocols is to reduce CNI exposure, by converting CNI to non-nephrotoxic immunosuppressive drugs with or without low dose CNI. MMF, and mammalian target of rapamycin (mTOR) inhibitors (mTORis), namely SRL and ERL, are the commonest options in CNI minimization protocols for their non-nephrotoxicity and potent immunosuppression effects [14]–[23].

In the current meta-analysis (32 controlled studies included with a total of 1383 patients), both GFR and sCr comparison presented a significant improvement of renal function in CNI minimization group in both RCTs and observational studies analysis. Although there was significant improvement of CrCl in CNI minimization over routine CNI regimen in observational studies, we could not document such improvement in RCTs analysis. According to the National Kidney Foundation recommendation, GFR is the best estimate of kidney function and is used in the NKF staging of CKD, thus it is rational to conclude that CNI minimization is capable to restore renal function of liver transplant patients with CNI-related renal impairment.

To explore whether CNI reduction is safe in liver transplant recipients, we performed a meta-analysis of acute rejection episodes, infection rates and patient survival. There was no significant difference in acute rejection episodes between CNI minimization and routine CNI regimen, suggesting the immunosuppression effect was not compromised in patients taking on CNI minimization. However, the incidence of infections is higher in CNI minimization group than in routine CNI group, although most studies did not provide the exact incidence of specific infections, suggesting increased immune load by CNI minimization protocols when introducing MMF or mTORis. On the other hand, there was no significant difference in patient survival between two groups, which can be interpreted in three ways. Firstly, the follow-up durations of these studies are not long enough to show any change of the survival rate. Secondary, the improvement in renal function is not sufficient enough to alter patient survival [45]. Finally, the renal benefit may be counteracted by the increased infection risks. Therefore, whether the improved renal function can be translated into a better survival and whether long term use of CNI minimization protocols would lead to a higher acute rejection or infection rate still need further study.

Notably, the CNI minimization protocols in the included studies are heterogenous. Since the additional drugs and different combinations can alter the outcomes, we divided them into 3 subgroups, namely MMF-based, SRL-based and ERL-based subgroups. In both meta-analysis of RCTs and observational trials, the MMF-based regimen presents an obvious benefit on renal function protection. It has been reported that replacement of CNI by MMF in liver transplant patients with renal dysfunction can also improve other CNI associated side-effects, such as hypertension and hyperuricemia [46]. Concern about this CNI minimization protocol is from the fact that there are conflicting results regarding the risk of allograft rejection with withdrawal of CNI and subsequent MMF monotherapy [14], [29], [47]–[50]. However, in the current meta-analysis, MMF-based CNI minimization protocol is not associated with higher rejection and infection rates. One of the major reasons explaining the discrepancy of rejection rate between the previous reports and current analysis is that most recent protocols are combination of MMF and low-dose CNI but not MMF monotherapy. Collectively, we recommend that MMF can serve as a good option to reduce CNI exposure in liver transplant recipients with renal dysfunction, without increasing rejection and infection rates.

In addition, mTORis are potent anti-proliferative agents that have clear therapeutic potential in liver transplantation [51]–[55]. However, only 4 SRL-based RCTs were included in this meta-analysis, and no significant improvement of renal function was documented, neither did the ERL-based RCTs. But in the meta-analysis of observational trials, SRL-based regimen yielded a significant improvement of renal function as shown in GFR and sCr, in spite of relatively high heterogeneities. Both SRL-based and ERL-based regimens are equally potent and safe as routine CNI regimen in immunosuppression according to our meta-analysis. Moreover, in non-renal dysfunction population, SRL, either used in combination with prednisone alone or MMF-prednisone in CNI-withdrawal protocols, resulted in improved renal function and acceptable acute rejection rate, although with increased rates of thrombocytopenia, digestive hemorrhage, pleural effusion and other adverse events [56]. In terms of ERL, Simone et al recently reported that ERL, in combination with low-dose CNI, was associated with low acute rejection rate and particularly good renal function [15]. However, in another study, the use of combination CsA and mTORis leaded to potential long-term CNI nephrotoxicity [57]. Since the number of SRL-based and ERL-based RCTs included in this meta-analysis is small, more high-quality RCTs based on SRL and ERL should be conducted to draw a clear conclusion on whether mTORis-based CNI minimization protocols are effective and safe in patients with impaired renal function. However, according to the results from the meta-analysis of observational trials and considering their well known anti-tumor effects [58]–[60], mTORis may be a good alternative for MMF to reduce or replace CNI in liver transplant recipients with a pre-transplant diagnosis of hepatocellular carcinoma (HCC) and post-transplant renal dysfunction. However, clinicians should pay attention to the increased risks of infections when SRL is used.

Undoubtedly, there are some limitations in the current meta-analysis as others. Firstly, we included studies using different regimens without comparing between themselves, it make us difficult to figure out which combination is the best one although the current data show that the MMF-based CNI minimization protocol received the greatest supports. Secondly, most of the studies we included didn't undertake follow-ups longer than 12 months, giving us insufficient data on how CNI minimization would affect long-term graft or patient survival. Finally, as shown in Table 2, the risk of bias of the included randomized trials was relatively high, since no study was double blind designed and only 3 of 10 studies conducted intention-to-treat analysis, which may attenuate the power of the current study.

In conclusion, this meta-analysis included all current relevant studies from various countries covering different populations. It can make up to the shortage of small sample size and limited population of individual studies, providing stronger evidence on the clinical application of CNI minimization protocols. It is convincing that CNI minimization can improve renal function in liver transplant patients with CNI-related renal impairment, while has an equal or similar effect on acute rejection and patient survival as routine CNI regimen. However, it should be cautious to use SRL-based minimization regimens in patients with high risks of infections. Studies in the future should try to figure out whether this improved renal function can prolong long-term patient or graft survival, and which minimization protocol is the standard one in various combinations.

## Supporting Information

Figure S1 **Meta-analysis of CNI minimization versus routine CNI regimen in GFR (observational trials).** In MMF and sirolimus subgroups, GFR was significantly higher than in the routine CNI regimen group, so was in the total analysis. Z = total effect size, I^2^ = heterogeneity index. Columns in green represent the mean difference of each study and column size represents the study weight in the analysis. Lanes represent the 95% CI of each study. Diamonds in black represent the overall effect size and diamond width represents the overall 95% CI.
(TIF)

Figure S2 **Meta-analysis of CNI minimization versus routine CNI regimen in sCr (observational trials).** In MMF, sirolimus and everolimus subgroups, the SCr was significantly decreased in CNI minimization group, so was in the total analysis. Z = total effect size, I^2^ = heterogeneity index. Columns in green represent the mean difference of each study and column size represents the study weight in the analysis. Lanes represent the 95% CI of each study. Diamonds in black represent the overall effect size and diamond width represents the overall 95% CI.
(TIF)

Figure S3 **Meta-analysis of CNI minimization versus routine CNI regimen in CrCl (observational trials).** In MMF subgroup, CrCl was significantly higher in the CNI minimization group than in the routine CNI regimen group. In everolimus subgroup, improvement of CrCl over routine CNI regimen group was not statistically significant. In the total analysis, the CrCl was significantly improved in CNI minimization group. Z = total effect size, I^2^ = heterogeneity index. Columns in green represent the mean difference of each study and column size represents the study weight in the analysis. Lanes represent the 95% CI of each study. Diamonds in black represent the overall effect size and diamond width represents the overall 95% CI.
(TIF)

## References

[1] Organ Procurement and Transplant Network http://optn.transplant.hrsa.gov/LatestData/viewDataReports.asp.

[2] Ojo AO, Held PJ, Port FK, Wolfe RA, Leichtman AB (2003). Chronic renal failure after transplantation of a nonrenal organ.. N Engl J Med.

[3] Lebron Gallardo M, Herrera Gutierrez ME, Seller Perez G, Curiel Balsera E, Fernandez Ortega JF (2004). Risk factors for renal dysfunction in the postoperative course of liver transplant.. Liver Transpl.

[4] Pawarode A, Fine DM, Thuluwath PJ (2003). Independent risk factors and natural history of renal dysfunction in liver transplant recipients.. Liver Transpl.

[5] Moreno JM, Cuervas-Mons V, Rubio E, Pons F, Herreros de TA (2003). Chronic renal dysfunction after liver transplantation in adult patients: prevalence, risk factors, and impact on mortality.. Transplant Proc.

[6] Campbell KM, Yazigi N, Ryckman FC, Alonso M, Tiao G (2006). High prevalence of renal dysfunction in long-term survivors after pediatric liver transplantation.. J Pediatr.

[7] Cantarovich M (2004). Renal dysfunction in liver transplantation: the problem and preventive strategies.. Can J Gastroenterol.

[8] Johnson DW, Saunders HJ, Johnson FJ, Huq SO, Field MJ (1999). Fibrogenic effects of cyclosporin A on the tubulointerstitium: role of cytokines and growth factors.. Exp Nephrol.

[9] Johnson DW, Saunders HJ, Johnson FJ, Huq SO, Field MJ (1999). Cyclosporin exerts a direct fibrogenic effect on human tubulointerstitial cells: roles of insulin-like growth factor I, transforming growth factor beta1, and platelet-derived growth factor.. J Pharmacol Exp Ther.

[10] Myers BD, Ross J, Newton L, Luetscher J, Perlroth M (1984). Cyclosporine-associated chronic nephropathy.. N Engl J Med.

[11] Jain A, Vekatramanan R, Eghtesad B (2005). Long-term outcome of adding mycophenolate mofetil to tacrolimus for nephrotoxicity following liver transplantation.. Transplantation.

[12] Kornberg A, Kupper B, Hommann M, Scheele J (2005). Introduction of MMF in conjunction with stepwise reduction of calcineurin inhibitor in stable liver transplant patients with renal dysfunction.. Int Immunopharmacol.

[13] Cantarovich M, Tzimas GN, Barkun J, Deschenes M, Alpert E (2003). Efficacy of mycophenolate mofetil combined with very low-dose cyclosporine microemulsion in long-term liver-transplant patients with renal dysfunction.. Transplantation.

[14] Schlitt HJ, Barkmann A, Boeker KHW, Schmidt HH, Emmanouilidis N (2001). Replacement of calcineurin inhibitors with mycophenolate mofetil in liver transplant patients with renal dysfunction: a randomized controlled study.. Lancet.

[15] Simone PD, Metselaar HJ, Fischer L, Dumortier J, Boudjema K (2009). Conversion from a calcineurin inhibitor to everolimus therapy in maintenance liver transplant recipients: a prospective, randomized, multicenter trial.. Liver Transpl.

[16] Cicinnati VR, Yu Z, Klein CG, Sotiropoulos GC, Saner F (2007). Clinical Trial: switch to combined mycophenolate mofetil and minimal dose calcineurin inhibitor in stable liver transplant patients - assessment of renal and allograft function, cardiovascular risk factors and immune monitoring.. Aliment Pharmacol.

[17] Beckebaum S, Cicinnati VR, Klein CG, Brokalaki E, Yu Z (2004). Impact of combined mycophenolate mofetil and low-dose calcineurin inhibitor therapy on renal function, cardiovascular risk factors, and graft function in liver transplant patients: preliminary results of an open prospective study.. Transplant Proc.

[18] Pageaux GP, Rostaing L, Calmus Y, Duvoux C, Vanlemmens C (2006). Mycophenolate mofetil in combination with reduction of calcineurin inhibitors for chronic renal dysfunction after liver transplantation.. Liver Transpl.

[19] Eisenberger U, Sollinger D, Stickel F, Burckhardt B, Frey FJ (2009). Relationship between renal resistance index and renal function in liver transplant recipients after cessation of calcineurin inhibitor.. Clin Transplant.

[20] Rogers CC, Johnson SR, Mandelbrot DA, Pavlakis M, Horwedel T (2009). Timing of sirolimus conversion influences recovery of renal function in liver transplant recipients.. Clin Transplant.

[21] Biselli M, Vitale G, Gramenzi A, Riili A, Berardi S (2009). Two yr mycophenolate mofetil plus low-dose calcineurin inhibitor for renal dysfunction after liver transplant.. Clin Transplant.

[22] Shenoy S, Hardinger KL, Crippin J, Desai N, Korenblat K (2007). Sirolimus conversion in liver transplant recipients with renal dysfunction: a prospective, randomized, single-center trial.. Transplantation.

[23] Beckebaum S, Klein CG, Stiropoulos GC, Saner FH, Gerken G (2009). Combined mycophenolate mofetil and minimal dose calcineurin inhibitor therapy in liver transplant patients: clinical results of a prospective randomized study.. Transplantation Proc.

[24] Pulido LB, Alamo Martinez JM, Pareja Ciuro F, Gomez Bravo MA, Serrano Diez-Canedo J (2008). Efficacy and safety of mycophenolate mofetil monotherapy in liver transplant patients with renal failure induced by calcineurin inhibitors.. Transplantation Proc.

[25] Reich DJ, Clavien PA, Hodge EE (2005). Mycophenolate mofetil for renal dysfunction in liver transplant recipients on cyclosporine or tacrolimus: randomized, prospective, multicenter pilot study results.. Transplantation.

[26] Benedetto FD, Sandro SD, Ruvo ND, Montalti R, Guerrini GP (2009). Immunosuppressive switch to sirolimus in renal dysfunction after liver transplantation.. Transplantations Pro.

[27] Ponton C, Vizcaino L, Tome S, Otero E, Molina E (2010). Improvement of renal function after conversion to mycophenolate mofetil combine with low-level calcineurin inhibitor in liver transplant recipients with chronic renal dysfunction.. Transplant Proc.

[28] Creput C, Blandin F, Deroure B, Roche B, Saliba F (2007). Long-term effects of calcineurin inhibitor conversion to mycophenolate mofetil on renal function after liver transplantation.. Liver Transpl.

[29] Koch RO, Graziadei IW, Schulz F, Nachbaur K, Konigsrainer A (2004). Long-term efficacy and safety of mycophenolate mofetil in liver transplant recipients with calcineurin inhibitor-induced renal dysfunction.. Transpl Int.

[30] Raimondo ML, Dagher L, Papatheodoridis GV, Rolando N, Patch DW (2003). Long-term mycophenolate mofetil monotherapy in combination with calcineurin inhibitors for chronic renal dysfunction after liver transplantation.. Transplantation.

[31] Planas JMM, Martinez VCM, Gonzalez ER, Cruz AG, Lopez-Monclus J (2004). Mycophenolate mofetil can be used as monotherapy late after liver transplantation.. Am J Transplant.

[32] Tannuri U, Gibelli NEM, Maksoud-Filho JG, Santos MM, Pinho-Apezzato ML (2007). Mycophenolate mofetil promotes prolonged improvement of renal dysfunction after pediatric liver transplantation: experience of a single center.. Pediatr Transpl.

[33] Pfitzmann R, Klupp J, Langrehr JM, Neuhaus R, Junge G (2002). Mycophenolate mofetil reduces calcineurin inhibitor-induced side effects after liver transplantation.. Transplant Proc.

[34] Fairbanks KD, Eustace JA, Fine D, Thuluvath PJ (2003). Renal function improves in liver transplant recipients when switched from a calcineurin inhibitor to sirolimus.. Liver Transpl.

[35] Sanchez EQ, Martin AP, Ikegami T, Uemura T, Narasimhan G (2005). Sirolimus conversion after liver transplantation: improvement in measured glomerular filtration rate.. Transplant Proc.

[36] Yang YJ, Chen DZ, Li LX, Kou JT, Lang R (2008). Sirolimus-based immunosuppressive therapy in liver transplant recipient with tacrolimus-related chronic renal insufficiency.. Transplant Proc.

[37] Orlando G, Balocchi L, Cardillo A, Laria G, Liguori ND (2007). Switch to 1.5 grams MMF monotherapy for CNI-related toxicity in liver transplantation is safe and improves renal function, dyslipidemia, and hypertension.. Liver Transpl.

[38] Simone PD, Precisi A, Petruccelli S, Balzano E, Carrai P (2009). The impact of everolimus on renal function in maintenance liver transplantation.. Transplant Proc.

[39] Dharancy S, Iannelli A, Hulin A, Declerck N, Schneck AS (2009). Mycophenolate mofetil monotherapy for severe side effects of calcineurin inhibitors following liver transplantation.. Am J Transplant.

[40] Morard I, Dumortier J, Spahr L, Hadengue A, Majno P (2007). Conversion to sirolimus-based immunosuppression in maintenance liver transplantation patients.. Liver Transpl.

[41] Vivarelli M, Dazzi A, Cucchetti A, Gasbarrini A, Zanello M (2010). Sirolimus in liver transplant recipients: a large single-center experience.. Transplantation Proc.

[42] Castroagudin J, Molina E, Varo E (2011). Proteinuria predicts unfavourable evolution after switching of immunosuppression from calcineurin-inhibitor to everolimus in liver transplant recipients with chronic renal dysfunction.. Journal of Hepatology.

[43] Cotterell AH, Fisher RA, King Al, Gehr TWB, Dawson S (2002). Calcineurin inhibitor-induced chronic nephrotoxicity in liver transplant patients is reversible using rapamycin as the primary immunosuppressive agent.. Clin Transplant.

[44] Campistol JM, Sacks SH (2000). Mechanisms of nephrotoxicity.. Transplantation.

[45] Lopez MM, Valenzuela JE, Alvarez FC, Lopez-Alvarez MR, Cecilia GS (2006). Long-term problems related to immunosuppression.. Transplant Immunology.

[46] Manzia TM, De Liguori Carino N, Oriando G, Toti L, De Luca L (2005). Use of mycophenolate mofetil in liver transplantation: a literature review.. Transplantation Proc.

[47] Stewart SF, Hudson M, Talbot D, Manas D, Day CP (2001). Mycophenolate mofetil monotherapy in liver transplantation.. Lancet.

[48] Moreno Planas JM, Cuervas-Mons MV, Rubio GE (2004). Mycophenolate mofetil can be used as monotherapy late after liver transplantation.. Am J Transplant.

[49] Fairbanks KD, Thuluvath PJ (2004). Mycophenolate mofetil monotherapy in liver transplant recipients: a single center experience.. Liver Transpl.

[50] Pierini A, Mirabella S, Brunati A, Ricchiuti A, Franchello A (2005). Mycophenolate mofetil monotherapy in liver transplantation.. Transplant Proc.

[51] McAlister VC, Peltekian KM, Malatjalian DA, Colohan S, MacDonald S (2001). Orthotopic liver transplantation using low-dose tacrolimus and sirolimus.. Liver Transpl.

[52] Watson CJ, Friend PJ, Jamieson NV, Frick TW, Alexander G (1999). Sirolimus: a potent new immunosuppressant for liver transplantation.. Transplantation.

[53] Chang GJ, Mahanty HD, Quan D, Freise CE, Ascher NL (2000). Experience with the use of sirolimus in liver transplantation - use in patients for whom CNIs are contraindicated.. Liver Transpl.

[54] Kovarik JM, Kahan BD, Kaplan B, Lorber M, Winkler M (2001). Longitudinal assessment of everolimus in de novo renal transplant recipients over the first post-transplant year: pharmacokinetics, exposure-response relationships, and influence on cyclosporine.. Clin Pharmacol The.

[55] Levy G, Schmidli H, Punch J, Tuttle-Newhall E, Mayer D (2006). Safety, tolerability, and efficacy of everolimus in de novo liver transplant recipients: 12- and 36-month results.. Liver Transpl.

[56] Watson CJE, Gimson AES, Alexander GJ, Allison MED, Gibbs P (2007). A randomized controlled trial of late conversion from calcineurin inhibitor (CNI)-based to sirolimus-based immunosuppression in liver transplant recipients with impaired renal function.. Liver Transpl.

[57] Lam P, Yoshida A, Brown K, Abouljoud M, Bajjoka I (2004). The efficacy and limitations of sirolimus conversion in liver transplant patients who develop renal dysfunction on calcineurin inhibitors.. Digestive Diseases and Sciences.

[58] Vivarelli M, Cucchetti A, Barba GL, Ravaioli M, Del Gaudio M (2008). Liver transplantation for hepatocellular carcinoma under calcineurin inhibitors: reassessment of risk factors for tumor recurrence.. Ann Surg.

[59] Panwalkar A, Verstovsek S, Giles FJ (2004). Mammalian target of rapamycin inhibition as therapy for hematologic malignancies.. Cancer.

[60] Guba M, Von Breitenbuch P, Steinbauer M, Koehl G, Flegel S (2002). Rapamycin inhibits primary and metastatic tumor growth by antiangiogenesis: involvement of vascular endothelial growth factor.. Nat Med.

