# Supplementary figures and images for: Calcineurin-Inhibitor Minimization in Liver Transplant Patients with Calcineurin-Inhibitor-Related Renal Dysfunction: A Meta-Analysis
Source: PLoS One. 2011 Sep 9;6(9):e24387. doi: 10.1371/journal.pone.0024387 (PMC3170329; doi:10.1371/journal.pone.0024387)

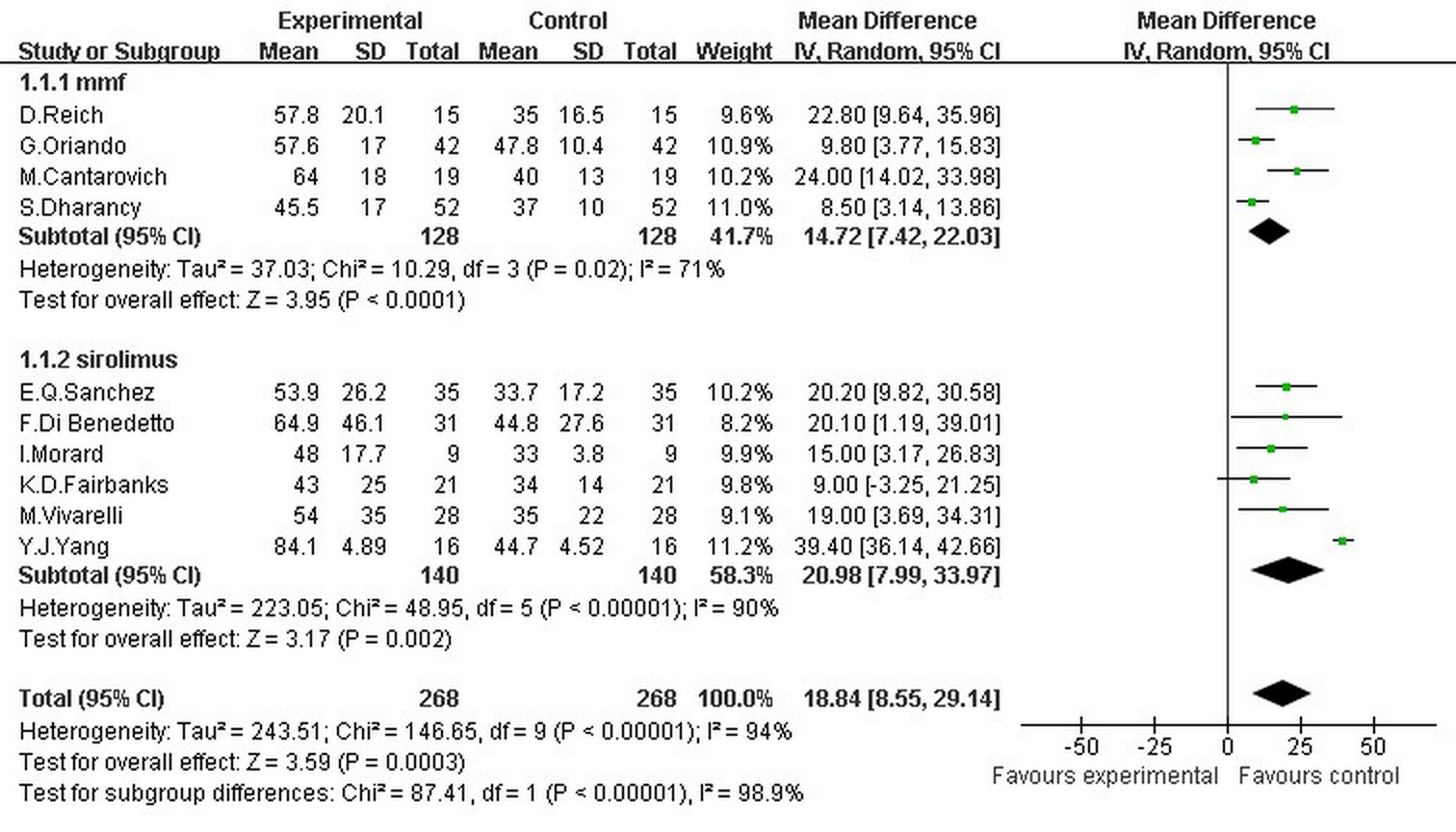

Supplement: Figure S1 — Meta-analysis of CNI minimization versus routine CNI regimen in GFR (observational trials). In MMF and sirolimus subgroups, GFR was significantly higher than in the routine CNI regimen group, so was in the total analysis. Z = total effect size, I2 = heterogeneity index. Columns in green represent the mean difference of each study and column size represents the study weight in the analysis. Lanes represent the 95% CI of each study. Diamonds in black represent the overall effect size and diamond width represents the overall 95% CI. (TIF) [file pone.0024387.s001.tif]

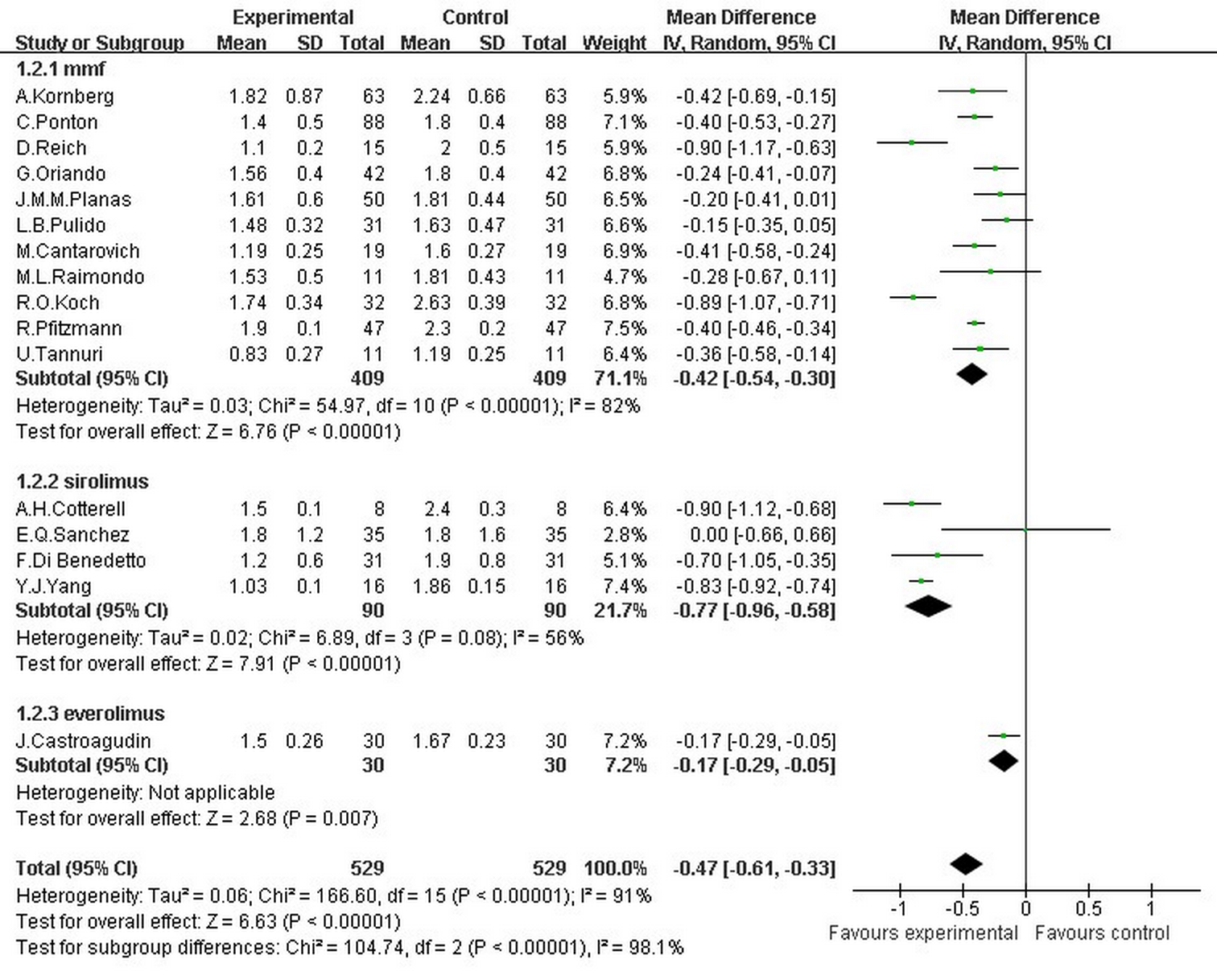

Supplement: Figure S2 — Meta-analysis of CNI minimization versus routine CNI regimen in sCr (observational trials). In MMF, sirolimus and everolimus subgroups, the SCr was significantly decreased in CNI minimization group, so was in the total analysis. Z = total effect size, I2 = heterogeneity index. Columns in green represent the mean difference of each study and column size represents the study weight in the analysis. Lanes represent the 95% CI of each study. Diamonds in black represent the overall effect size and diamond width represents the overall 95% CI. (TIF) [file pone.0024387.s002.tif]

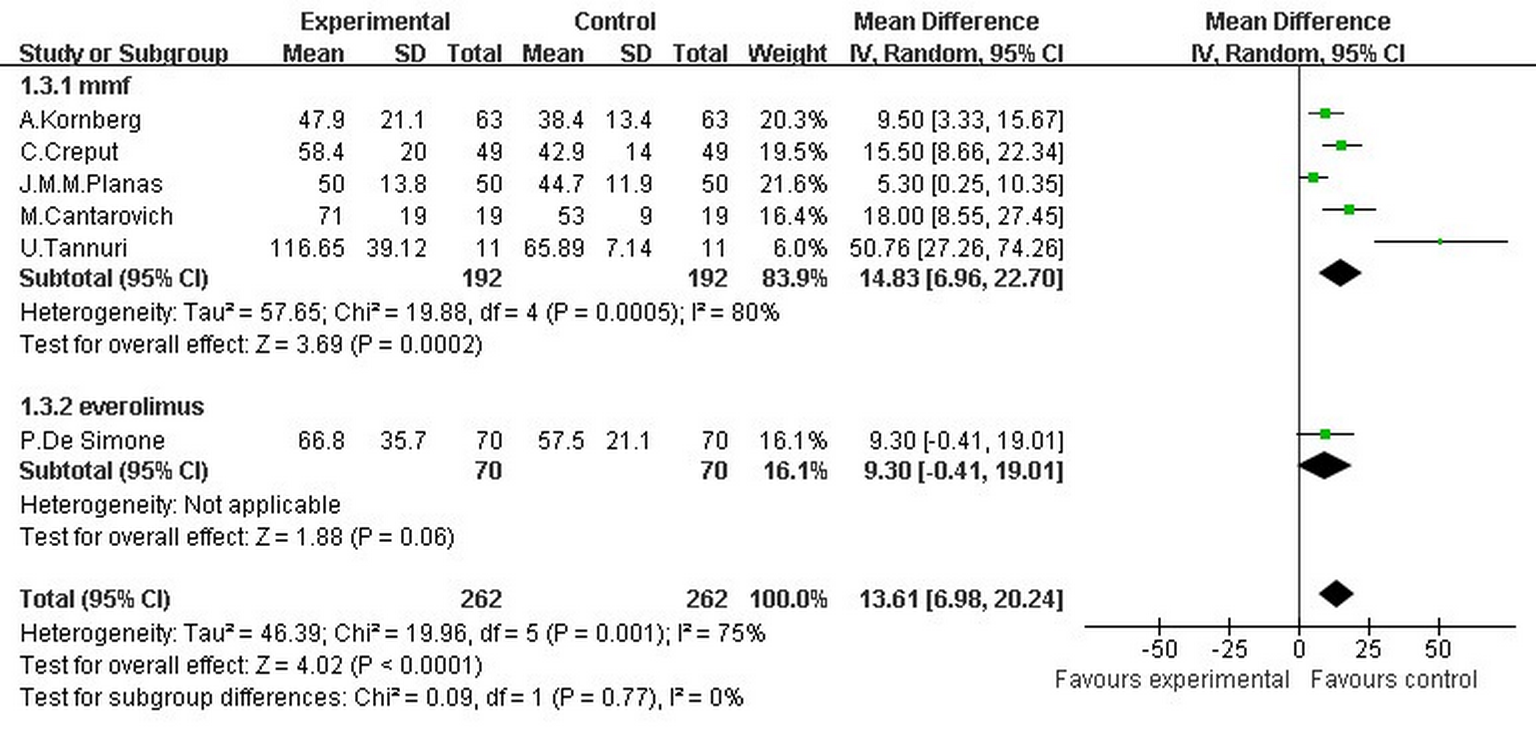

Supplement: Figure S3 — Meta-analysis of CNI minimization versus routine CNI regimen in CrCl (observational trials). In MMF subgroup, CrCl was significantly higher in the CNI minimization group than in the routine CNI regimen group. In everolimus subgroup, improvement of CrCl over routine CNI regimen group was not statistically significant. In the total analysis, the CrCl was significantly improved in CNI minimization group. Z = total effect size, I2 = heterogeneity index. Columns in green represent the mean difference of each study and column size represents the study weight in the analysis. Lanes represent the 95% CI of each study. Diamonds in black represent the overall effect size and diamond width represents the overall 95% CI. (TIF) [file pone.0024387.s003.tif]
